# Supplementary material for: Examination of 2D frontal and sagittal markerless motion capture: Implications for markerless applications
Source: PLoS One. 2023 Nov 9;18(11):e0293917. doi: 10.1371/journal.pone.0293917 (PMC10635560; doi:10.1371/journal.pone.0293917)
Supplement: S1 Table — (DOCX) [file pone.0293917.s004.docx]

S3 Table

Table 1: Repeat measures Bland-Altman analysis of 2D markerless ankle, knee and hip joint centre locations in the sagittal and frontal plane, relative to reprojected marker-based motion capture. Values are normalised to the vertical torso height of the participant, taken from the marker-based motion capture reprojected values, averaged between the distances of the left hip-shoulder and right hip-shoulder. Camera-side represents the right side of the body which was closest to the camera, while Occluded-side represents the left side of the body which was furthest from the camera in the sagittal view. Left and right sides of the body were combined in the frontal plane. All values were normalised to the vertical distance in pixels (height) between the left and right shoulder and hip joint centre locations of the participant, for every frame in each individual trial.

| Joint Locations | | Bias  (Norm Pixels) | SD of Bias  (Norm Pixels) | LOA  (Norm Pixels) |
| --- | --- | --- | --- | --- |
| Sagittal Plane | | | | |
| MTP | **Camera-side** | 0.117 | 0.052 | 0.015 – 0.219 |
|  | **Occluded-side** | 0.135 | 0.061 | 0.015 – 0.255 |
| Ankle | **Camera-side** | 0.052 | 0.041 | -0.028 – 0.132 |
|  | **Occluded-side** | 0.052 | 0.044 | -0.034 – 0.138 |
| Knee | **Camera-side** | 0.070 | 0.040 | -0.008 – 0.148 |
|  | **Occluded-side** | 0.064 | 0.038 | -0.010 – 0.138 |
| Hip | **Camera-side** | 0.05 | 0.031 | -0.011 – 0.111 |
|  | **Occluded-side** | 0.088 | 0.040 | 0.010 – 0.166 |
| Shoulder | **Camera-side** | 0.049 | 0.018 | 0.014 – 0.084 |
|  | **Occluded-side** | 0.068 | 0.037 | -0.005 – 0.141 |
| Frontal Plane | | | | |
| MPT | | 0.055 | 0.029 | -0.002 – 0.112 |
| Ankle | | 0.047 | 0.027 | -0.006 – 0.100 |
| Knee | | 0.057 | 0.027 | 0.004 – 0.110 |
| Hip | | 0.055 | 0.022 | 0.012 – 0.098 |
| Shoulder | | 0.074 | 0.017 | 0.041 – 0.107 |
