# Supplementary material for: Examination of 2D frontal and sagittal markerless motion capture: Implications for markerless applications
Source: PLoS One. 2023 Nov 9;18(11):e0293917. doi: 10.1371/journal.pone.0293917 (PMC10635560; doi:10.1371/journal.pone.0293917)
Supplement: S1 Fig — (DOCX) [file pone.0293917.s005.docx]

S4 Figures


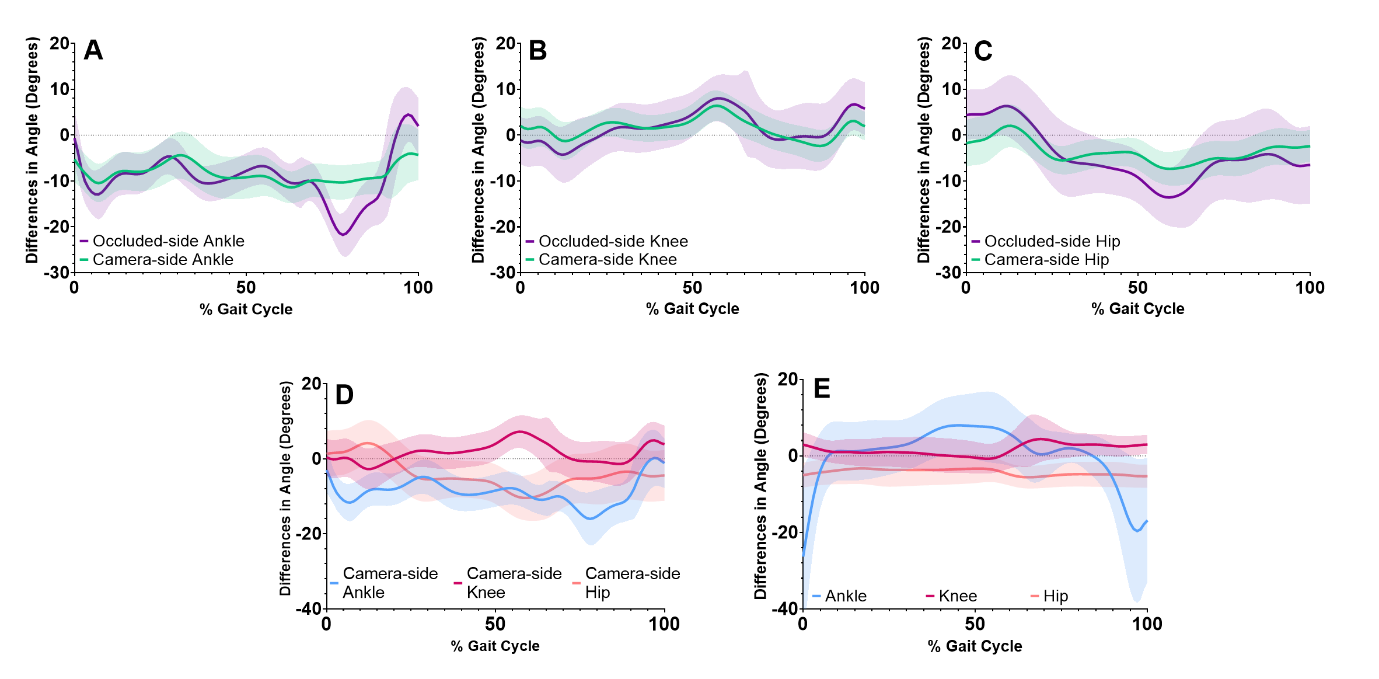


Figure 1: Markerless motion capture joint angle differences, relative to marker-based motion capture, of the ankle, knee and hip. Graph A-C compares the occluded and camera-side joints for the sagittal plane ankle (A), knee (B) and hip (C). Graph D compares the camera-side joints of the ankle, knee and hip in the sagittal plane. Graph E compares the joint angles of the ankle, knee and hip in the frontal plane (left and right angles have been combined).


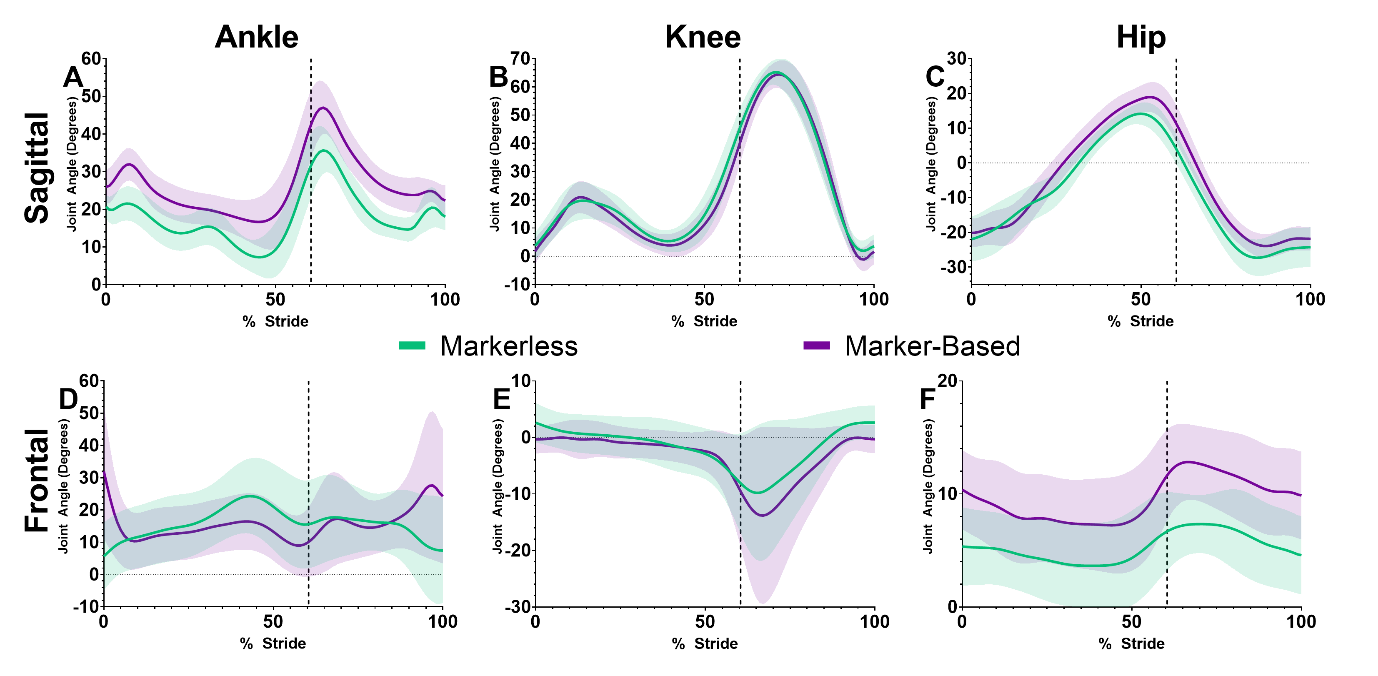


Figure 2: Comparison of joint angles (mean ± standard deviation) between markerless and marker-based motion capture in the sagittal (A,B,C) and frontal plane (D, E, F) for the ankle (A, D), knee (B, E) and hip (C, F). Sagittal plane joints are the camera-side joints only.
