# Supplementary material for: Examination of 2D frontal and sagittal markerless motion capture: Implications for markerless applications
Source: PLoS One. 2023 Nov 9;18(11):e0293917. doi: 10.1371/journal.pone.0293917 (PMC10635560; doi:10.1371/journal.pone.0293917)
Supplement: Attachment — Submitted filename: Reply to Reviewers - V8.docx [file pone.0293917.s006.docx]

We would like to thank all reviewers for their valuable insight into this manuscript and believe the changes made to the manuscript have improved the value and strength of this work.

**Editor Comments**

A limitations section needs to be added.

REPLY: We have added a limitations section to the manuscript

Please ensure that your manuscript meets PLOS ONE's style requirements, including those for file naming. The PLOS ONE style templates can be found at 
<https://journals.plos.org/plosone/s/file?id=wjVg/PLOSOne_formatting_sample_main_body.pdf> and 
<https://journals.plos.org/plosone/s/file?id=ba62/PLOSOne_formatting_sample_title_authors_affiliations.pdf>

TO DO: Done

We note that the grant information you provided in the ‘Funding Information’ and ‘Financial Disclosure’ sections do not match. When you resubmit, please ensure that you provide the correct grant numbers for the awards you received for your study in the ‘Funding Information’ section.

REPLY: The grant numbers in the funding information are correct. We are not sure what this is referring to. We have included an updated financial disclosure (which may have been the issues) in the cover letter as suggested in the comments email. If this not correct, please let us know more specifically what the issue is and can resolve this.

Please include captions for your Supporting Information files at the end of your manuscript, and update any in-text citations to match accordingly. Please see our Supporting Information guidelines for more information: <http://journals.plos.org/plosone/s/supporting-information>.

REPLY: Captions for Supporting information have been added to the paper and naming of these files have been updates in the caption and in-text citations

**Reviewer 1 Comments**

In the results, the authors should describe that the limits of agreement according to the Bland and Altman plots are wide, which shows a significant error or bias for the detection of ankle angle, but also exists for the hip joint.

REPLY: We adjusted a sentence in the results highlighting the poor limits of agreement in the occluded hip joint (LINE 210).

We believe that this was also performed in the first discussion paragraph (LINES 263-265), through relating this error to the total ROM of the joint. While the absolute limits of agreement appear high in the hip joint relative to the ankle angle, the higher ROM experienced at the hip joint means that the signal-to-noise ratio of this joint is much lower than the ankle which is the key outcome measure.

We also highlighted this on LINES 282-284

LINE 210:

However, the Bland-Altman limits of agreement ranges (random differences) were 40%, 69% and 108% greater in the occluded ankle, knee and hip respectively, compared to their camera-side counterparts (Table 1, Fig 3A-C).

LINE 263-265:

Sagittal plane random differences of occluded joint angles were higher in more proximal joints, with the hip being highest (Table 1, Fig 3). Because occluded-side hip and shoulder joint centre location were obstructed for the entire stride, they were more affected than the MTP, ankle and knee (Fig 2).

LINE 282-284:

Frontal plane joint angle systematic differences were relatively small for the ankle and knee, while the hip exhibited a larger offset (Fig 3), although this is most likely due to the systematic differences previously identified in this markerless training dataset (16).

The discussion is correct and does a detailed analysis of the results, showing the limitations of OpenPose and describing alternatives. I recommend that the authors make recommendations for clinical staff.

REPLY: We have added text to the paragraph below in the discussion to better highlight our recommendations.

Line 323-327

Our findings suggest that for clinical, sporting and research applications during walking, 2D sagittal and frontal plane kinematics identified by the OpenPose algorithm could be obtained for the visible knee and hip joint angles during stance, the sagittal ankle angle is likely only usable during early and mid-stance, and the frontal plane ankle angle is too variable across the whole stride.

**Reviewer 2 Comments**

There are some issues with the use of correct terminology for accuracy/precision/reliability.

TO DO: We have re-edited the manuscript to be consistent with our terminology throughout the manuscript

Some methodological details are missing and the paper lacks a discussion of limitations of their own work.

TO DO: We have addressed all comments listed below and believe that we have filled in this methodological detail and have added a limitations section of the paper.

Keywords: consider adding “markerless motion capture” if possible

REPLY: The purpose of a keyword section is to include search terms that are not found in the title (which are already keywords). Because the term ‘markerless motion capture’ is included in the title of the paper, it therefore does not need to be a keyword

Title – Would recommend “Examination of 2D frontal and sagittal markerless motion capture” as the title.

REPLY: We have chosen to keep the original title as we believe this paper has direct implications for both 2D and 3D markerless motion capture, as such, including this in the title is important to outline the paper as a whole and make finding our paper easier for researchers who are searching for this information.

Line 23 - Please be more specific about the markerless motion capture system used (i.e. 2D markerless motion capture from a single camera view).

REPLY: We have updated this sentence to better describe the markerless motion capture system used as suggested.

LINE 23:

This study examined if occluded joint locations obtained from 2D markerless motion capture (single camera view) produced 2D joint angles with reduced accuracy compared to visible joints, and if 2D frontal plane joint angles were usable for practical applications.

Line 35 – are these only marker-based methods?

TO DO: It is probably more specific to state that these are methods that are using marker-based data. As such, this sentence has been reworked to improve clarity.

Line 36:

As such, this paper finishes by reviewing methods for creating anatomically accurate markerless training data using marker-based motion capture data.

Starting the paper with issues with marker-based motion capture seems sub-optimal for a paper that has a primary aim of comparing markerless against a marker-based reference. This paper may be better motivated without the initial discussion of marker-based problems, which would be more appropriate in a limitations section. Additionally, the authors could note that while marker-based motion capture may not necessarily accurately reflect the motion of the underlying skeletal anatomy, as the aim of the first objective was to compare visible vs occluded 2D markerless motion capture, 3D marker-based is expected to perform equally well on both sides, enabling the markerless occluded vs visible comparison.
Furthermore, the discussion of the lack of access to x-ray-based methods to calculate markerless accuracy may not be helpful. These approaches still have errors. This discussion implies that markerless systems that do not have equivalent “accuracy” to x-ray-based methods are not useful, which I’m guessing is not the intent.

REPLY: Thank you for this comment. This was not the intent and we agree that this would be better if moved to a limitations sections and thus have done so.

LINE 410:

While this study has compared the accuracy and variability of markerless motion capture against the 3D marker-based method, inherent marker-based errors such as soft tissue artefact (25, 26), marker placement (33, 36) and altered participant movement patterns (37) will obscure the markerless results. As such determining the accuracy of markerless systems is difficult due to scarcity and complexity of gold standard methods such as bi-planar radiographic imaging (23, 48) and videoradiography (26, 49). This should not have affected our comparison between occluded and visible joint centres, however the absolute accuracy and variability we observed may differ from that obtained against gold standard methods.

Line 39 – Please consider if “accuracy and reliability” are the correct terms here and throughout the paper (see further comments on accuracy for Lines 77-78, 80)

TO DO: Thank you for pointing this out. As outlined in their 2007 paper, Bland-Altman highlight that the limits of agreement inform the variability of a measure, not the reliability. As such, we have re-edited the manuscript to improve consistency of our terms and ensure that language used throughout the manuscript relates better to accuracy informed by bland-altman bias and variability informed by the bland-altman standard deviation of the bias/limits of agreement.

Bland, J. M., & Altman, D. G. (2007). Agreement Between Methods of Measurement with Multiple Observations Per Individual. J Biopharm Stat, 17(4), 571-582. doi:10.1080/10543400701329422

Line 50 - Please provide further motivation for the prospective use of single camera markerless motion capture beyond “without the need for specialist personnel to process the data”

REPLY: When rearranging the introduction, this sentence has been combined with a previous sentence and therefore this comment is no longer relevant to the new text.

Lines 52-56 – The wording here implies that the 2 studies mentioned were using an algorithm that identifies occluded joint locations, but that does not appear to be the case. It is not entirely clear why these 2 studies are mentioned (are these the only studies that have reported sagittal plane kinematics using a single camera 2D markerless system?) or why you discuss different algorithms than those used by these 2 studies.

REPLY: You are correct and this sentence incorrectly implies that these two studies could detect the occluded joint. We have amended this sentence by splitting it in two and adding additional references that highlights the point we were intending to make. Thank you for highlighting this for us.

Line 46:

Previous 2D markerless motion capture research has examined sagittal plane kinematics during overground and treadmill walking, and underwater running (4, 6, 11, 12), identifying joint angles errors that are in line with those obtained when using manually labelled methods or marker-based motion capture. However, to date, only angles of joints nearest to the camera have been examined (11, 12), despite pose estimation algorithms often providing joint centre locations on the occluded far side of the body (13).

Line 61 – please remove “inevitable”

REPLY: we have removed ‘inevitable’ from this sentence

Lines 68 – 69 – “low random differences” from what? Again, consider the correct terms to use (if precision and reliability are intended to mean the same thing, standardize your terminology)

REPLY: Thank you for pointing out this inconsistent use of terminology. We have standardised this terminology with that used in the previous paragraph.

Line 62:

In the 2D frontal plane, joint occlusion is minimal, but limited movement requires high accuracy and low variability to detect small but meaningful changes.

Line 77 – 78 – Reconsider the use of the term “accurate” in your objectives since you argue that your reference technology (marker-based) is not accurate and therefore you lack a true value against which to measure.

REPLY: We have expanded these aims to specifically relate to marker-based motion capture. While we do believe that marker-based motion capture has inherent errors, the original manuscript appears to have over-emphasised this, based on your comments. We believe with these changes here, and with the marker-based errors being moved to the limitations section, this should correct this.

Line 80 – Define here or in the methods your definitions of “too variable” and “meaningful changes” against which your results are compared or otherwise define the threshold for acceptance of “accurate” markerless kinematic results. Note that “too variable” indicates a lack of precision, not a lack of accuracy.

REPLY: This has been added to the methods

LINE 169:

Insufficient accuracy or variability of the markerless system were defined as differences relative to marker-based methods that are greater than known marker-based errors previously reported (25-27).

The further objective of discussing methods of creating improved markerless training data is missing.

REPLY: This has been added.

LINE 81:

Finally, this paper will finish by reviewing methods for creating anatomically accurate markerless training data (labelled images) using marker-based motion capture data.

Please define acronyms (i.e. MTP, TTL) at the first instance

REPLY: These have been defined.

Line 86 – How was constant speed achieved? Was it the same for all participants?

REPLY: This was preferred constant speed walking, therefore the preferred speed was different for each participant. This was omitted from the methods section and thus has been added. Thank you for highlighting.

LINE 183:

The average preferred walking speed across all participants was 1.55 ± 0.23 m/s

Lines 111 – 133 - The details of the tracking method to identify the participant across multiple frames would be appropriate for a supplement.

REPLY: We are cautious of having too many supplementary papers as these should really only be used when necessary. As the details of the tracking method are less than one paragraph, we believe it makes sense to keep it in the main portion of the manuscript.

Line 142 – 145 - Visual3D does not provide recommendations for joint location approaches, but several options. Please indicate the approaches used and reference the original papers for the chosen approach.

REPLY: Apologies for this oversimplification. This sentence has been expanded which should clarify these issues.

Line 142:

Hip joint centres were computed via regression from the anterior and posterior superior iliac spine markers (20), knee and ankle joint centres were computed as the midpoint between the medial and lateral markers, shoulder joint centres were inferiorly offset from the acromioclavicular joint shoulder markers by 2.5 cm, and the MTP joint centres were computed as the midpoint between the first and fifth MTP joint markers.

Line 152 – Replace “high-quality” with “aligned” or similar

REPLY: ‘High quality’ has been replaced with ‘aligned’.

Results: Specify the number of trials and strides recorded and how many were included in analysis.
Were any data lost due to problems with marker-based or markerless tracking?

REPLY: This has been added to the manuscript

LINE 181:

Each participant completed 10 preferred speed walking trials for a total of 150 trials (15 participants x 10 trials). However due to marker-drop out, or incorrect foot placement (i.e., the entire foot was not placed on the force plate), 130 successful trials were analysed.

Please report the walking speed.

REPLY: Average preferred walking speed across participants has been recorded

LINE 183

The average preferred walking speed across all participants was 1.55 ± 0.23 m/s

Differences in pixels are not very meaningful as they depend on the camera resolution and the distance of the participants to the cameras, neither of which are provided.

REPLY: Resolution has been added to the methods (LINE 94). The primary reason that we have reported joint centre location errors in pixels is because in the frontal plane, participants are walking towards the camera and are thus changing their distance to the camera not only in every single frame, but joint locations relative to other joints are also different within each frame. For example, at certain points in the stride, the right foot will be much closer to the camera than the left foot. Secondly, we are primarily comparing results between joints to understand why differences in joint angles exist. Thus, the primary outcome measure is joint angle, the pixel location errors simply serve to further explain these differences. Therefore, to maintain continuity through all outcome measures, it made sense to report pixel location differences.

Line 201 – The term “absolute random differences” is not meaningful. Why not compare the limits of agreement?

REPLY: We found the standard deviation of bias easier to understand however it would appear the limits of agreement ranges might be easier to understand. Therefore, this has been renamed and we recalculated the results to report LOA as a percentage. However, because the absolute random differences (standard deviation of the bias) and limits of agreement ranges are based on the same distribution, these values are identical.

LINE 210:

However, the Bland-Altman limits of agreement ranges (random differences) were 40%, 69% and 108% greater in the occluded ankle, knee and hip respectively, compared to their camera-side counterparts (Table 1, Fig 3A-C).

Line 272 – “the relative proportion of error to signal is lower.” Should this be: “higher” instead of lower? (higher proportion of error relative to signal)

REPLY: Thank you for correcting this error. We have replaced ‘lower’ with ‘higher’

Line 290 – unclear how the conclusion of “discounting occluded joint results” is reached when there has been no definition of acceptability.

REPLY: This has now been added to the methods section as suggested in a previous comment

LINE 169:

Insufficient accuracy or variability of the markerless system were defined as differences relative to marker-based methods that are greater than known marker-based errors previously reported (25-27).

Line 311 – I would encourage the authors to consider whether equivalent accuracy to biplanar x-ray is the goal of markerless or if the data may still be useful if it doesn’t not achieve the same resolution. Further, having a reference methodology that is less prone to issues such as soft tissue movement does not eliminate differences due to differing coordinate system definitions.

REPLY: We are not suggesting that markerless systems will not be acceptable if they do not achieve the same resolution as biplanar x-ray. We are highlighting that marker-based methods have known errors, and therefore we are unable to truly identify the accuracy of markerless methods if we only compare them the marker-based methods. We have adjusted this sentence to improve clarity and emphasise this point.

We do agree that comparing to biplanar fluoroscopy will not overcome differing coordinate systems definitions, hence the need for the final paragraphs exploring ways to improve labelled data. We have added a sentence to this paragraph to this effect.

LINE 321:

Due to these known marker-based errors, comparisons between marker-based motion capture and markerless motion capture give us a strong estimate of the accuracy and variability of markerless motion capture. Our findings suggest that for clinical, sporting and research applications during walking, 2D sagittal and frontal plane kinematics identified by the OpenPose algorithm could be obtained for the visible knee and hip joint angles during stance, the sagittal ankle angle is likely only usable during early and mid-stance, and the frontal plane ankle angle is too variable across the whole stride. To determine the true accuracy and variability of markerless systems, comparisons against gold standard measures such as bone pins and biplanar fluoroscopy may be required, although differing coordinate system definitions may still exist between systems that could result in systematic differences.

Line 318 – Be specific in your discussion of implications for 3D motion capture systems. Are these statements true for all 3D markerless systems or only the ones you are familiar with?

REPLY: This sentence has been expanded to better specify the markerless systems to which we are referring.

LINE 332:

The results from this study also have implications for 3D markerless motion capture systems that are trained on open access dataset, due to observed difference between occluded and visible (camera-side) joint centre locations.

Line 336 – 354 – Unclear of the relevance of this discussion to the current paper.

REPLY: Thank you for this comment. We were conflicted if this section should be included and based on your comment, we agree that this paper would likely be enhanced and more focussed with it removed.

Line 351 – 353 – Would caution against predicting the results of another study without analysis of the data.

REPLY: This section has been removed

Line 425: Unclear what is meant by “loosen data setup restrictions common to 2D motion capture.”

REPLY: This section has been removed as the paragraph it was referring to has been removed (see previous comments)

There are no limitations of your study discussed. Limitations of marker-based motion capture would be appropriate in such a section. Please include discussion of limitation of clothing. In clinical applications, it is unlikely the participants will be wearing minimal spandex. Please also discuss limitations related to the B&A approach (From Bland JM & Altman DG. 2007: “It is an implicit assumption that the difference between the two methods is reasonably stable across the range of measurements” – Is that true for this study?)
Please include limitations of sample size and a young healthy sample
Unlikely to have force data in clinical/sporting applications. How would the use of kinematic	gait events affect the data?

REPLY: We have added a limitations section which discusses marker-based limitations, clothing and our young healthy sample (Line 409-426

The limitations of the bland altman analysis are included in the supplementary file (last paragraph) which we feel is more appropriate, given that this document has a much more detailed overview of the bland altman method.

There are a number of studies that have demonstrated that a force plate is not necessary to extract gait event data from markerless kinematic data. We used force plate data in this study to ensure that gait events were detected at the exact same time between both marker-based and markerless systems. We have expanded this section of the methods to highlight this point.

LINE 409-426:

Limitations

While this study has compared the accuracy and variability of markerless motion capture against the 3D marker-based method, inherent marker-based errors such as soft tissue artefact (25, 26), marker placement (33, 36) and altered participant movement patterns (37) will obscure the markerless results. As such determining the accuracy of markerless systems is difficult due to scarcity and complexity of gold standard methods such as bi-planar radiographic imaging (23, 48) and videoradiography (26, 49). This should not have affected our comparison between occluded and visible joint centres, however the absolute accuracy and variability we observed may differ from that obtained against gold standard methods. While our study had participants wearing tight clothing so that marker-based motion capture could be used, this is generally not possible in clinical environments. However, recent work has demonstrated that the Thiea markerless system is able to handle day-to-day clothing and even baggy pants relatively well (50), which should also hold true for the OpenPose pose estimation algorithm, and thus, day-to-day clothing should not alter our results substantially. As outlined in our S1 Appendix, this study made a comparison of 13231 data points (130 trials x 101 data points) which is more than sufficient to make comparisons between motion capture methods. However, it should be noted that data was collected on relatively young healthy adults, and thus these results may be restricted when examining population groups that differ substantially from our participants (e.g. overweight participants, amputees or children).

Figure 4 A – plot is truncated

REPLY: This plot has been reformatted to fix this and remove repeating text

Table 1 – define LOA

REPLY: this abbreviation was not used enough throughout the text and therefore, we have simple said limits of agreement when talking about this.

Supplement 1 – should this be referring to Supplement 2 (not 3) for tables, data, etc?

REPLY: Yes, thank you very much for finding this error, we have corrected this

Supplement 2 - unclear what the sample data represents (i.e. are these joint angles? Which joint?)

REPLY. This has been added to the title of each sheet in the excel supplementary file.

Supplementary File 2 (now called S2 Data)

‘Example of repeat measures Bland-Altman formula and common-sense testing performed on the occluded ankle joint angle in the sagittal plane’

Supplement 3 Figure 1 – please label vertical axis as “Differences in Angle”

REPLY: The y axis has been altered to Differences in Angle

Supplement 3 Figure 1E – unclear why the L/R sides are compared

REPLY: The left and right sides were not compared, this was unclear from the text. The text has been updated to say

‘Graph E compares the joint angles of the ankle, knee and hip in the frontal plane (left and right angles have been combined).’
