# Supplementary material for: Examination of 2D frontal and sagittal markerless motion capture: Implications for markerless applications
Source: PLoS One. 2023 Nov 9;18(11):e0293917. doi: 10.1371/journal.pone.0293917 (PMC10635560; doi:10.1371/journal.pone.0293917)
Supplement: Attachment — Submitted filename: Reply to Reviewers_V10.docx [file pone.0293917.s007.docx]

We would like to thank all reviewers for their valuable insight into this manuscript and believe the changes made to the manuscript have improved the value and strength of this work.

**Editor Comments**

The methods part shall be more detailing to reflect how you carry out the testing

We have added detail to the manuscript to better reflect how the testing was carried out.

**Reviewer 2 Comments**

The authors still seem to be missing correct definitions of terms critical to their study, specifically accuracy.

In measurement terms, accuracy refers to how close measured values are to “true” values. As true values are difficult to determine in many cases, the Bland & Altman approach was developed specifically to compare 2 measurement systems (to determine their interchangeability, i.e. limits of agreement) in the absence of true values. As such, the Bland & Altman method cannot be used to assess accuracy. The authors can choose to (1) define marker-based motion capture results as the true values and then calculate the accuracy of the markerless system OR (2) they can use the Bland & Altman approach to compare the offset and limits of agreement between markerless and marker-based motion systems, but in this case they cannot report accuracy.

REPLY: We must apologies for incorrectly interpreting this comment in the first round of reviews. We believed this comment related to inconsistent use of terms, but now better understand the reviewers comment. We agree with the reviewer that given the issues of marker-based methods, the use of the term accuracy is incorrect. Therefore, we have replaced the word accuracy with agreement/difference/bias where necessary throughout the manuscript

"Lines 75 – 80 - Reconsider the use of the term “accurate” in your objectives since you argue that your reference technology (marker-based) is not accurate and therefore you lack a true value against which to measure.

REPLY: This has now been changed

Line 80 - Note that “too variable” indicates a lack of precision, not a lack of accuracy."

REPLY: This has now been changed

They should also stick with the terms defined by Bland & Altman (limits of agreement) instead of using “random difference” which will not be meaningful to most readers.

REPLY: We have changed random difference to standard deviation of bias or limits of agreement throughout the manuscript

Literature discussed in the introduction remains subpar. The inclusion of articles that did not measure frontal joint angles is confusing.

REPLY: While we believe that the inclusion of these two papers do add to this section, given that there are so few papers that have performed markerless motion capture in the frontal plane, the reviewer clearly has strong feelings about this. As such, we have removed this sentence and reworked this paragraph based on comments from other reviewers.

Basic methodological pieces are missing, i.e. recording frequencies, image resolutions, if joint locations defined in the same way for both systems.

REPLY: All of this information was included in the manuscript already. Video data was recorded at 200 Hz (Line 113). Image resolution of the machine vision cameras were 1920x1080 (Line 114). Joint location definitions were listed on Lines 190-192. Because markerless data was not trained on the marker-based joint centre locations, these are different. This is a known limitation, which has been discussed in many previous papers, and is something we had also discussed here, although we have expanded this section to make this clearer (Line 315-319)

The methods of how front plane joint positions are measured in both systems are not described.

REPLY: This was described in great detail in the paragraph starting Line 163 and specifically line 166 and 169. This was also outlined in detail in Figure 1.

The authors have not justified reporting absolute differences in pixels, which seems like it will not be meaningful, as the size of the joint will change in every frame as the participant moves further from or closer to the camera.

REPLY: To account for this, all pixel differences have now been normalised to the averaged vertical distance in pixels (height) between the left and right shoulder and hip joint centre locations, obtained from the markerless motion capture system. Averaged left and right, hip and shoulder distances were used as these joint locations remain in the approximately the same plane as the centre of mass for both frontal and sagittal plane movements. It should be noted that while this method will overall minimise errors as they get closer to the camera, it does not account for the left and right limbs being closer or further away from the camera at different points in the stride. However, because we asked participants to hit the first force plate with their right foot five times, and then they alternated by hitting the first force plate with their left foot five times, this should not affect one side of the body more than the other. While this normalisation was not likely needed for the sagittal plane, it will help to minimise any parallax error and allow comparisons in the error magnitude between the sagittal and frontal planes. This explanation and rationale have been added to the manuscript Line (192). These changes had no effect on the discussion as the main result trends were identical with and without the correction. Below you will find the absolute pixel locations figure and the normalised pixel locations figures for the frontal plane. You will see that this method minimises the differences between systems at the end of the stride that you correctly suggested would occur. This method also fixes differences at the end of the stride, which were higher than he beginning of the stride, which would not be expected to occur in a cyclic measurement. Thank you for highlighting this. Because we are unable to report the distance of the participant to the camera to give context to the absolute pixel differences, we have elected to only include the normalised pixel values in the manuscript.

Calculations of joint angles and percentages are missing from the methods.

REPLY: Joint angle calculation was described on line 190 and in Figure 1. Percentage differences of the joint angle bias, relative to the joint range of motion (measured from the marker-based motion capture system) has been added to the methods (Line 228)

Figure 2 sub plots are not identified

REPLY: Apologies, this was actually included, however, figure 3 was incorrectly uploaded as figure 2, and figure 2 was incorrectly uploaded as figure 3. This should be fixed now that figures have been uploaded correctly.

Figure captions are incomplete (i.e. lines and shaded regions indicate what?)

REPLY: This has been added to the caption

I maintain that the title is an overreach. It is unclear how your conclusions apply to 3D analysis.

REPLY: We have adjusted the title

**Reviewer 3**

The study offers important insights into the strengths and limitations of markerless motion capture, and specifically sheds light on its limitations in accurately capturing occluded joint angles, particularly in the sagittal plane. In addition, here are some other potential issues that should be considered for the next revision

In the introduction section, it would be beneficial to provide a brief overview of the background and current status of 2D and 3D marker-based motion capture techniques, and explain why these techniques are crucial in the analysis of human motion. Additionally, it is suggested to further clarify the research purpose and research questions to help readers better understand the motivation and value of this paper.

REPLY: There have been a number of highly in-depth reviews on the background and current status of 2D and 3D marker-based motion capture techniques. As such we have added a sentence that directs readers to these reviews. (Line 43).

We have added to the introduction to further highlight the potential of markerless motion capture and how it may be crucial for the future development and analysis of human movement. (Lines 46-54)

We have also added a sentence to the introduction which further clarifies motivation for the research, especially with respect to examination of 2D frontal measures. (Line 74)

It would be easier for readers to understand if you could explain the difference between occluded joints and visible joints.

REPLY: This has been added to Line 60.

It is recommended to describe the specific algorithms or techniques used for markerless 2D and 3D motion capture, ensuring sufficient details are provided to allow readers to understand your experimental setup and data collection process.

We have added additional detail to the methods to better describe the methods used (Lines 118-124, Line 192-208).

In the discussion part, it is recommended to provide a brief description of the aim and main findings in the first paragraph of the manuscript

REPLY: While we respect the personal preference of the reviewer to start the discussion in this format, we believe this comment has no impact on the scientific merits of this study. The addition of this paragraph would not improve the scientific merits of this manuscript, and therefore our preference is to not include this in the discussion, as it simply repeats what is already written in the introduction, results and discussion.

In the conclusion part, please also show relevant descriptions about the contributions for future clinical or scientific research.

REPLY: The conclusion has been reworked to better describe how this work contributes to clinical and scientific research (Line 473)

Furthermore, the future research direction and prospects are also an important part of the article, and it would be beneficial to expand on this section and provide more detailed content.

REPLY: The discussion section has been expanded to include this content (Line 482).
