# Supplementary material for: Examination of 2D frontal and sagittal markerless motion capture: Implications for markerless applications
Source: PLoS One. 2023 Nov 9;18(11):e0293917. doi: 10.1371/journal.pone.0293917 (PMC10635560; doi:10.1371/journal.pone.0293917)
Supplement: Attachment — Submitted filename: Reply to Reviewers - V11.docx [file pone.0293917.s008.docx]

**Reviewer 3 Comments**

No Comments

**Reviewer 4**

In this work, the authors investigated the accuracy of 2D joint angles obtained from occluded joint locations in markerless motion capture compared to visible joints. The author recruited 15 participants to recorded while walking using marker-based and machine vision cameras. The results showed that markerless joint angles on the occluded side had higher variation and disagreement compared to visible joints. However, the visible joint angles in the sagittal plane were relatively accurate and consistent with marker-based measurements. On the frontal plane, the markerless joint angles had acceptable agreement with marker-based data for the hip and knee but not for the ankle. The study also identified limitations in current pose estimation algorithms and datasets, suggesting the need for improved methods to create anatomically accurate markerless training data using marker-based motion capture. This work could be accepted after minor revisions. Other questions were shown below.

REPLY: We thank the reviewer for taking the time to read our manuscript. We believe that we have addressed all the reviewer comments and the clarity of the manuscript is now further improved. Please note that all line changes referred to below are from the tracked changes document.

The study only included 15 healthy participants, which is a relatively small sample size. A larger sample size can improve the reliability and generalization ability of the results

REPLY: We agree with the reviewer that the findings from 15 healthy participants may not be widely applicable to other populations, e.g., clinical populations, and certainly more work is needed in the future to ensure that the same agreement can be achieved in those populations. This was touched on in the limitations section, however this section has been expanded to further describe the limitations of only including 15 healthy participants. Line 452-455. Nonetheless, we do feel that the estimates of agreement presented in this paper based on this sample do provide a very useful addition to the literature upon which future research can build.

This study used indoor marker cameras and machine vision cameras for data collection, which may differ from outdoor or actual application scenarios. Therefore, more exploration and validation are needed for applications in different environments.

REPLY: We fully agree that further exploration and validation is needed in less controlled scenarios and with lower than ‘research grade’ technology. However, a first key step was to ensure that the markerless system can work comparably to marker-based in a laboratory-based setting. We have added this to the limitations section. Line 440-446

This study mainly uses the Bland Altman analysis to evaluate the consistency, but other metrics are also worth considering, such as correlation coefficient or Root-mean-square deviation. Combining multiple evaluation indicators can provide a more comprehensive analysis of results. Have other statistical analyses been conducted to evaluate the reliability and significance of the results, in addition to the Bland Altman analysis?

REPLY: We used a repeat measures Bland-Altman analysis, as the standard bland-Altman analysis assumes all data points are independent, which is not true for our dataset that has repeated measures. This is a similar assumption for standard correlation coefficient and root-mean-square deviation analysis. As such to use these methods, the data generally needs to be averaged multiple times to meet this assumption, drastically reducing the variability. However, we do agree that other metrics are worth considering, thus we have included a repeated measures correlation statistical analysis to Table 1, which provides a correlation coefficient value between marker-based and markerless motion capture for repeated measures joint angles. This analysis is not possible to calculate for pixel locations, given that the normalised resultant pixel differences we report are made up of a 2D pixel locations (x and y coordinates). It would not make sense to break down the resultant pixel locations into their 2D values for these repeated measures correlation analysis alone. Methods for the repeat measures correlation coefficient have been added to the methods (Line 208-210), and implications of these results has been added throughout the results and discussion sections.

Can we further explain or discuss why the unmarked deviation standard deviation and consistency limit of the occluded joint on the Sagittal plane are twice that of the visible joint? May this difference be related to the characteristics of pose estimation algorithms?

REPLY: This has already been discussed at length on Lines 62-65 356-360, 376-381

In the discussion, can we explore the shortcomings of current pose estimation algorithms and labeled datasets, and propose suggestions for improvement or future research directions?

REPLY: This is a crucial aspect of the paper, which was already explored in depth on Lines 356-431

Considering that the consistency limit of ankle joint is still higher than the requirements of practical application, can you provide suggestions or ideas on how to optimize unmarked Motion capture of ankle joint？

REPLY: Without improving the labelling in markerless datasets, this will be a challenging task. We have outlined methods for potentially improving these datasets in Lines 375-431. We have also added a sentence to the discussion on lines 320-323 which outlines a potential short-term solution that fuses markerless motion capture with IMU devices to improve accuracy at the ankle.
